# Conserved Cell-Type-Specific Transcriptomic Networks and Regulatory Programs Underlie Alcohol Dependence Across Mouse and Human

**DOI:** 10.21203/rs.3.rs-9417955/v1

**Published:** 2026-04-21

**Authors:** Nihal A. Salem, Anna S. Warden, Amanda J. Roberts, Marisa Roberto, R. Dayne Mayfield

**Affiliations:** (1) Waggoner Center for Alcohol and Addiction Research, The University of Texas at Austin, Austin, TX 78712, USA; (2) Department of Neuroscience, The University of Texas at Austin, Austin, TX 78712, USA; (3) Animal Models Core Facility, The Scripps Research Institute, La Jolla, CA, 92073, USA; (4) Department of Translational Medicine, The Scripps Research Institute, La Jolla, CA, 92073, USA;

## Abstract

Alcohol use disorder (AUD) is a complex polygenic disease. Rodent models of alcohol dependence have been instrumental in modeling various aspects of dependence. Single-nucleus transcriptomics has enabled the profiling of cell-type-specific changes in gene expression in both human AUD and animal models.

In this study, we identified shared dysregulated transcriptomic networks (TN), comprising gene co-expression modules and gene regulatory networks (GRNs) in a mouse model of alcohol dependence and individuals with AUD. Through cell-type-specific TN analysis, we identified translationally relevant, conserved dependence dysregulated molecular signatures.

We identified conserved dependence-upregulated gene co-expression modules in astrocytes and oligodendrocytes, with hub genes Slc1a3 and Pde4b, respectively. These genes are linked to alcohol dependence mechanisms, such as glutamate signaling, a well-established target of alcohol’s effects, and PDE4, whose inhibition has been shown to reduce alcohol intake in preclinical and clinical studies. We then integrated publicly available human and mouse GRN data to identify upstream regulators of alcohol-dysregulated gene signatures in each cell type. This approach revealed a set of transcription factors (TFs), including Mef2a, Mef2c, Jund, Nr3c1, and Zeb1, that were upstream of most dysregulated genes in both the mouse and human datasets and have established relevance to addiction biology, representing promising targets for translational research.

Collectively, these findings demonstrate the utility of cross-species, cell-type-specific network analysis for uncovering conserved molecular mechanisms in alcohol dependence. The identification of shared dysregulated networks, cell type homology, and upstream regulators provides a foundation for developing translationally relevant targeting strategies that can be tested in animal models.

## Introduction:

Alcohol use disorder (AUD) is a complex polygenic disease characterized by dysregulated gene expression and neurobiological adaptations. Genome-wide association studies (GWAS) and transcriptomic analyses have identified numerous genes associated with escalated alcohol consumption. However, genes function within interconnected transcriptomic networks that regulate diverse cellular processes and adaptations. Identifying dysregulated networks and modules enables a systems-biology approach to understanding AUD as a multigene disorder characterized by intricate gene interactions.

Gene regulatory networks (GRNs) represent the interactions between genes and their upstream transcriptional regulators. Integrating gene expression and chromatin accessibility data provides a systems-level perspective on transcriptional control and regulatory architecture. Within these networks, genes with correlated expression patterns can be clustered into gene co-expression modules.

Integrated analyses of bulk transcriptomes from mouse models of alcohol dependence, GWAS, and human protein-protein interactions have identified alcohol-responsive and dependence-associated gene networks in the prefrontal cortex (PFC) and other brain regions^1^. Cross-species network comparisons in cocaine exposure models have identified conserved gene co-expression modules, hub genes, and upstream regulators involved in addiction-relevant processes^2,3^. Network analysis of microarray transcriptomics from the PFC of mice and monkeys chronically exposed to ethanol revealed a conserved, dysregulated myelin-related gene co-expression network^4^. A similar approach identified estrogen-related receptor gamma (ERRγ) as a master regulator contributing to the PFC gene expression signature associated with a history of alcohol dependence in individuals with AUD; targeting this gene reduced alcohol consumption in multiple rodent models of alcohol dependence^5^. However, bulk tissue analyses obscure cell type-specific contributions to network dysregulation. Cell type-specific transcriptional regulation is critical for elucidating cellular mechanisms underlying complex brain disorders.

Recent advances in single-nucleus transcriptomics and GRN analysis have enabled the identification of cell type-specific dysregulated networks in aging and psychiatric disorders^6^. Emerging evidence highlights cell type-specific gene expression changes in alcohol dependence and withdrawal^7–11^, with functional studies revealing distinct cellular responses and roles in alcohol dependence^12–14^. However, cell type-specific GRNs and their role in addiction, including alcohol dependence, remain understudied. Identifying conserved, targetable gene networks dysregulated in both animal models and individuals with AUD is crucial for developing translationally relevant treatments.

Despite these advances, cell-type-specific GRNs in animal models and individuals with AUD remain poorly characterized. Accurate identification of disease-associated GRN dysregulation requires paired chromatin accessibility and transcriptomic profiling from the same cells in both control and disease conditions. In the absence of such paired data, upstream regulators can be approximated by leveraging GRNs derived from control tissue to infer TFs upstream of dysregulated transcriptomic signatures; however, this approach cannot capture AUD-specific alterations in network structure or TF-target relationships.

The goal of this study was to identify cell-type-specific dysregulated transcriptomic networks and key transcriptional regulators shared between a mouse model of alcohol dependence^9^ and postmortem brain samples from individuals with AUD^11^. Cross-species transcriptomic and regulatory network analyses were integrated to uncover conserved mechanisms with translational relevance underlying AUD and define novel avenues for therapeutic intervention.

## Methods:

### Datasets utilized:

Single-nucleus RNA sequencing (snRNA-seq) was performed on both a mouse model of alcohol dependence^9^ and postmortem brain samples from individuals with AUD^11^. The mouse dataset included medial prefrontal cortex (mPFC) samples from alcohol-dependent and control mice generated using the chronic intermittent ethanol (CIE) paradigm (~150,000 nuclei; GEO accession: GSE233763). The human dataset comprised snRNA-seq data from the dorsolateral prefrontal cortex (dlPFC) of individuals with AUD and matched controls (~500,000 nuclei; GEO accession: GSE247416). For anchor-based integration, the human dataset was subsampled to match the mouse dataset size. Cell type annotations, gene co-expression networks, and differential expression results were imported from previously annotated and constructed networks based on the original full datasets as described in^9,11^

### Anchor-based integration of mouse and human datasets.

**Figure 1:**
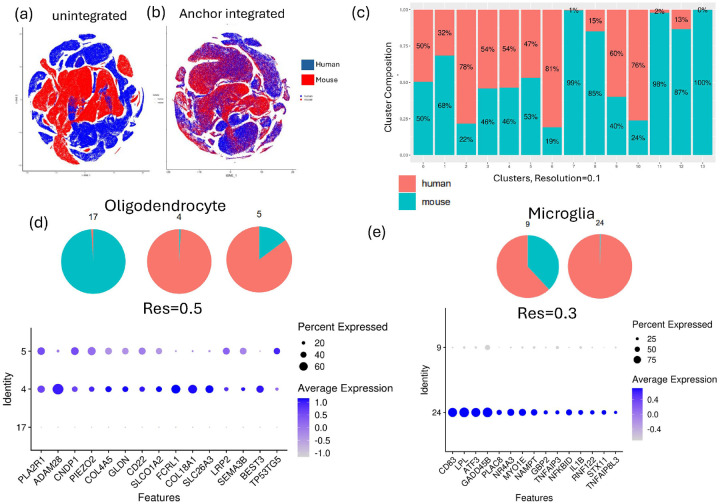
Anchor-based integration of mouse and human snRNA-seq datasets. t-SNE plot of **(a)** unintegrated and **(b)** anchor-integrated mouse (red) and subsampled human datasets (blue). **(c)** The percentage of mouse (orange) and human (green) cells in each cluster identified at resolution = 0.1. **(d)** Oligodendrocyte and **(e)** microglial clusters with species-specific contributions. top panel: Pie charts show the proportions of mouse (orange) and human (green) cells in each cluster; bottom panel: Dot plots display the top markers differentiating the subclusters.

Mouse CIE and human AUD datasets were integrated at the whole-transcriptome level using an anchor-based integration pipeline^15^ on the count matrices from the mouse dataset and the subsampled human dataset (described above) to identify homologous and species-specific cell types (Figures 1a and 1b). We used the “IntegrateData” function in Seurat (V5.0.2). Briefly, the top 2,000 variable genes across the mouse and human datasets, computed using FindVariableFeatures with the default “vst” feature selection, were selected as integration anchors. Canonical correlation analysis (CCA) was used to identify a shared subspace between the two datasets, followed by the identification of mutual nearest neighbors (MNNs) across datasets to define integration anchors. Anchor scores were computed to assess local neighborhood consistency, and correction vectors were applied using these anchors. This allowed for the projection of the two datasets into a common space while preserving the unique biological states present only in one of the datasets (Figures 1a and b). After integration was performed, unsupervised clustering using the default Louvain algorithm was conducted at multiple resolutions (0.1–0.8, in increments of 0.1). We used the Clustree package to visualize the origin of each cluster at multiple resolutions. The contribution of cells from each group (mouse control, mouse alcohol-dependent, human control, and human AUD) to each unsupervised cluster was calculated. Treatment-specific or species-specific clusters were identified at each resolution, as defined by clusters containing more than 60% of the cells from one species or one treatment. Genes enriched in each cluster were computed using FindAllMarkers using cutoff of log2 fold change > 0.25 and adjusted p-value < 0.01.

### Construction of consensus gene co-expression modules

We investigated differentially regulated gene co-expression modules identified in the mouse and human datasets^9,11^, and identified modules of interest which (1) share common hub genes, (2) show significant overlap in their gene members as determined by a hypergeometric test, and (3) show alcohol-dependence differential expression. Differentially expressed modules were determined by comparing module eigengene values between experimental groups (e.g., alcohol-dependent vs. control) using the default statistical testing in hdWGCNA. Module eigengenes, representing the first principal component of the expression matrix for each module, were tested for significant differences between conditions. Modules showing statistically significant differences in eigengene expression (Wilcoxon rank-sum test, adjusted p-value < 0.05) were classified as differentially expressed. We then utilized hdWGCNA^16^ to construct consensus modules in mouse and human glial cells. Mouse and human gene count matrices were combined, normalized, and metacells were constructed grouping by cell type, sample, and species. Metacells were then log-normalized, and networks were constructed using a soft power threshold of 0.8. Module eigengenes and connectivity were calculated, and genes with the highest intramodular connectivity values were designated as hub genes for each module. Modules, and their corresponding hub genes, were extracted. Module functions were investigated using gene ontology enrichment analysis via the GO Biological Process database in Enrichr, with the gene members of each module provided as input.

### Utilizing publicly available GRN information to identify upstream regulators of alcohol DEGs in mouse and human.

#### (a) Mouse

To identify upstream regulators of the mouse CIE-DEGs, we utilized publicly available gene regulatory networks (GRNs) and chromatin accessibility information provided by the NIH’s Brain Research through Advancing Innovative Neurotechnologies (BRAIN) Initiative - Cell Census Network (BICCN;^17^). In BICCN, GRNs were constructed by integrating single-nucleus ATAC-seq data (snATAC-seq, mapping accessible chromatin regions across ~2.3 million cells from 117 mouse brain regions) with matched single-cell RNA-seq profiles. Candidate cis-regulatory elements (cCREs) defined from snATAC-seq were linked to genes based on proximity (within 500 kb of transcription start sites) and positive correlation between accessibility and gene expression (FDR < 0.01). Putative enhancer-gene relationships were further refined using co-accessibility analyses (Cicero), using as a proxy for 3D chromatin interactions, to identify regulatory pairs. TF motifs were scanned within cCREs, and TF–gene regulatory edges were inferred when a TF motif was present in an enhancer linked to a gene. Machine learning approaches (ridge regression via CellOracle) were used to estimate TF influence and construct GRNs for over 260 cell subclasses.

For each cell type in our study, we identified the corresponding cell type in the BICCN data and imported the reported GRN information, including the list of target genes downstream of each TF. We then identified TFs upstream of each CIE-DEG and quantified the number of CIE-DEGs regulated by each TF, highlighting those TFs upstream of the largest number of CIE-DEGs (Figure 3a).

#### (b) Human

To identify upstream regulators of DEGs in human AUD (AUD-DEGs), we leveraged cell–type–specific gene regulatory networks (GRNs) from the prefrontal cortex, as reported in^6^ (https://brainscope.gersteinlab.org/integrative_files.html, files formatted as [celltype]_GRN.txt). These GRNs were constructed by integrating multiple data modalities: snRNA-seq and snATAC-seq data, TF motif enrichment analysis, co-accessibility analyses, gene co-expression, and single-cell QTL (scQTL) data from healthy individuals. For each cell type, candidate cis-regulatory elements (cCREs) were identified via chromatin accessibility profiling, and TF binding sites were predicted by motif enrichment analysis. Co-accessibility between cCREs and gene promoters, along with positive correlation between TF and target gene expression, was used to infer TF–gene regulatory edges.

For each cell type in our study, we imported the corresponding [celltype]_GRN.txt file, identified TFs upstream of each AUD-DEG, and quantified the number of AUD-DEGs regulated by each TF. We then identified TFs upstream of the largest number of AUD-DEGs and determined overlapping TFs that were upstream of the majority of both CIE-DEGs (mouse) and AUD-DEGs (human) (Figure 3a).

## Results:

### Identification of Convergent and Divergent Cell Types Between Mouse and Human

To systematically identify convergent and divergent cell types between mouse and human prefrontal cortex, we performed anchor-based integration of snRNA-seq datasets. The top 2,000 variable genes across both species were selected as integration anchors using Seurat’s FindVariableFeatures function. After integration, unsupervised clustering was performed across multiple resolutions to identify convergent cell types and detect species- or condition-specific subpopulations. At the lowest clustering resolution (0.1), we observed robust convergence between mouse and human astrocytes, oligodendrocytes, and microglia. Specifically, cluster 3 comprised the majority of astrocytes from both species, cluster 2 included oligodendrocytes, and cluster 9 contained microglia (Figure 1c). In contrast, clusters 7, 11, 12, and 13 at this resolution represented mouse-specific excitatory and inhibitory neuronal populations with no homologous clusters in human, highlighting species-specific divergence in certain neuronal subtypes.

At a higher clustering resolution (0.3), we identified a human AUD-specific microglial cluster (96% human AUD microglia) with no homologous mouse counterpart (Figure 1e). This cluster comprised disease-associated microglia and border-associated macrophages, and its marker genes were enriched for immune system and cytokine signaling pathways (CD83, ATF3, GADD45B, PLAC8, NR4A3, NAMPT, GBP2, TNFAIP3, NFKBID, IL1B), suggesting a unique human microglial response to alcohol dependence not recapitulated in the mouse model.

Subclustering of oligodendrocytes revealed a human-specific oligodendrocyte cluster, distinguished by significant enrichment of genes including PLA2R1, ADAM28, CNDP1, PIEZO2, COL4A5, GLDN, CD22, SLCO1A2, FCRL1, COL18A1, SLC26A3, LRP2, SEMA3B, BEST3, TP53TG5, and VIPR2 (Figure 1d). This finding is consistent with previous reports of human-specific acceleration and divergence of gene expression in oligodendrocytes^18^ and species-specific regulation of the VIPR2 promoter region^19^.

### Conserved Gene Co-Expression Modules Exhibit Alcohol-Mediated Dysregulation Across Mouse and Human Glial Cell Types.

We utilized hdWGCNA to identify alcohol-dysregulated co-expression modules in each cell type in the mouse and human datasets^9,11^. Modules of interest were defined as those that (1) shared common hub genes, (2) showed significant overlap in their gene members (assessed by a hypergeometric test), and (3) displayed alcohol-dependence differential expression. To investigate conserved molecular programs, we constructed mouse-human consensus co-expression modules within cell types shared across species, aiming to identify modules that mediate similar biological processes.

#### Astrocytes

**Figure 2:**
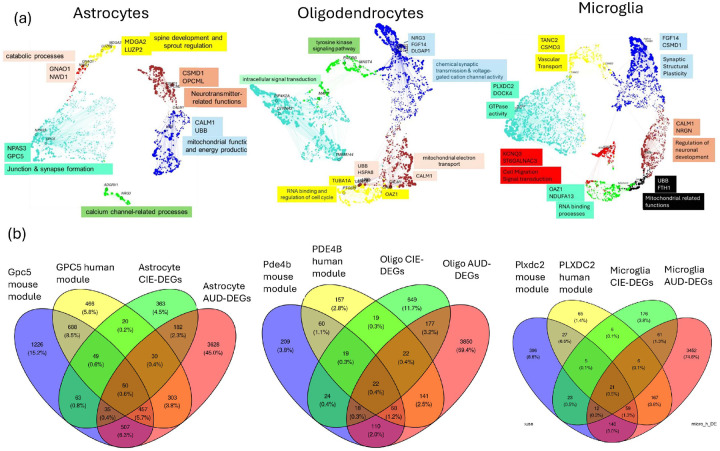
Conserved gene co-expression modules **(a)** UMAP visualization of consensus mouse-human hdWGCNA modules identified in astrocytes, oligodendrocytes, and microglia. Each dot represents a gene, with dot size scaled by the gene’s module membership score (kME) for its assigned module. Nodes represent genes, and edges represent co-expression relationships. Modules are annotated with top hub genes and representative enriched biological functions. **(b)** Venn diagrams showing the overlap between mouse and human differentially expressed genes (DEGs) and hdWGCNA modules of interest in mouse and human astrocytes, oligodendrocytes, and microglia.

hdWGCNA identified six mouse-human consensus modules in astrocytes, with functions categorized as follows: (1) ATP synthesis, mitochondrial function, and energy production (hub genes: Calm1, Ubb); (2) junction and synapse formation (hub genes: Npas3, Gpc5, Slc1a3); (3) neurotransmitter-related functions (hub genes: Csmd1, Opcml, Nrxn3); (4) spine development and sprout regulation (hub genes: Luzp2, Mdga2, Ptprt); (5) catabolic processes (hub genes: Nwd1, Gnao1, Phlg1); and (6) calcium channel-related processes (hub genes: Adgrv1, Nrg3, Ryr3) (Figure 2a). Consensus modules, gene members, hub genes, and functional categories are presented in **Supplementary Table 1**.

In mouse chronic intermittent ethanol (CIE) and human AUD astrocytes, hdWGCNA identified five and four modules, respectively. Mouse Astro-M3 and human Astro-M2 modules were upregulated in alcohol-exposed groups, shared hub genes, and exhibited significant gene membership overlap. Top hub genes included Gpc5, Nfia, Slc1a3, Npas3, and Prex2. Fifty genes overlapped between the hdWGCNA Gpc5 mouse and human modules and DEGs in astrocytes from mouse CIE and human AUD (Figure 2b). These overlapping genes are involved in processes such as disruption of postsynaptic signaling (CAMK2G, GRIN2C, TJP1, NRXN1, GRID1, GRM3, HRH1, SLC1A3, CHRDL1, GNG12, PHKA1, AKT2, SH3PXD2B, PRKD1, PTK2, DST, VCL, **Supplementary Table 2**), regulation of transsynaptic signaling.

CAMK2G, GRID1, GRIN2C, GRM3, HRH1, SLC1A3, NRXN1, CHRDL1), glutamatergic synapses (GRIN2C, GRM3, SLC1A3, GNG12), and cell projection morphogenesis (CDH4, CTNND2, GLI3, PTK2, PTPRZ1, VCL, NRXN1, PREX2). A comprehensive list of overlapping genes, Metascape analysis, and functional categories is presented in **Supplementary Table 2**.

#### Oligodendrocytes:

We identified five mouse-human consensus modules in oligodendrocytes, with hub genes and functions as follows: (1) chemical synaptic transmission and voltage-gated cation channel activity (hub genes: DLGAP1, FGF14, NRG3); (2) mitochondrial electron transport (hub genes: UBB, HSPA8, CALM1); (3) RNA binding and cell cycle regulation (hub genes: TUBA1A, PTGDS, OAZ1); (4) intracellular signal transduction (hub genes: TMEM144, PIP4K2A, CTNNA3); and (5) transmembrane receptor protein tyrosine kinase signaling pathway, and regulation of neuron projection development and axonogenesis (hub genes: MAST4, MAPT, PACRG) (Figure 2a). Consensus modules, gene members, hub genes, and functional categories are presented in **Supplementary Table 1**.

hdWGCNA identified ten modules in mouse oligodendrocytes and five in human oligodendrocytes. Mouse Oligo-M5 and human Oligo-M4 modules were upregulated in alcohol-exposed groups, shared hub genes (Pde4b and St18), and exhibited significant overlap in their gene members. Twenty-two genes overlapped between mouse and human modules and were differentially expressed in oligodendrocytes in both datasets. All 22 genes were upregulated in CIE mouse oligodendrocytes; in human AUD oligodendrocytes, nine were upregulated in Oligo1 subcluster (a cluster with myelination genes signature) and fifteen in Oligo3 subcluster (a cluster with inflammatory markers signature) (Figure 2b). These overlapping 22 genes are involved in CDC42 (FNBP1, DOCK10, FMNL2, DOCK1) and RHO GTPase cycles (DOCK1, FNBP1, DOCK10, FMNL2), pathways essential for proper myelination^20^, and cell-cell junction assembly (UGT8, CDH20, CDH19). A comprehensive list of overlapping genes, Metascape analysis, and functional categories is presented in **Supplementary Table 2**.

#### Microglia:

Consensus mouse-human microglial modules represented the following functional categories: (1) vascular transport (hub genes: TANC2, CSMD3, LYSMD4); (2) synapse structural plasticity and glutamate receptor signaling (hub genes: FGF14, CSMD1, NRG3, OPCML); (3) neuron projection development (hub genes: CALM1, NRGN, CKB, STMN1); (4) mitochondrial ATP synthesis and electron transport (hub genes: UBB, FTH1, CALM2, HSPA8); (5) GTPase activity and intracellular signal transduction (hub genes: DOCK4, PLXDC2, DOCK8, SRGAP2); (6) cell migration and signal transduction (hub genes: KCNQ3, ST6GALNAC3, ADAM28, SH3RF3); and (7) post-translational protein modification, transcription regulation, and cytokine signaling (hub genes: OAZ1, NDUFA13, COX5A, TPT1). Consensus modules, gene members, hub genes, and functional categories are presented in **Supplementary Table 1**.

hdWGCNA identified ten modules in mouse microglia and eight in human microglia. Five human microglial modules and three mouse microglial modules were upregulated in alcohol-exposed groups (Figure 2a); two mouse modules and two human modules were downregulated. Mouse Micro-M1 and human Micro-M1 modules, as well as mouse and human alcohol dysregulated microglial genes, shared thirteen overlapping genes. These genes are involved in the regulation of synaptic transmission (GRIK2, RASGRF2, NALCN, NTNG1, KCNJ3) and neuron projection morphogenesis (ROBO2, NTNG1, CNTNAP2, EPHA6, PCDH15). This transcriptional signature likely reflects microglial programs governing synaptic surveillance, pruning, and structural plasticity. Mouse M8 and human M5 modules, along with alcohol dysregulated microglial genes, shared 21 genes, the overlapping genes are involved in the regulation of cell migration (CMKLR1, P2RY12, DOCK8, ZFHX3, ITPR2, RAD51B) and cell activation (ENTPD1, DOCK2, BLNK, P2RY12, DOCK8, SRGAP2) (Figure 2b). Human Micro-M5 and mouse Micro-M8 shared Plxdc2 as a hub gene, and both overlapped with mouse-human consensus module 5, which is involved in GTPase activity and intracellular signal transduction. Human Micro-M1 and mouse Micro-M1 modules shared Fam155a as a hub gene, corresponding to consensus module 2, involved in synaptic plasticity and signal transduction. A full list of overlapping genes, Metascape analysis, and functional categories is presented in **Supplementary Table 2**.

### Gene Regulatory Networks in Alcohol Dependence

**Figure 3:**
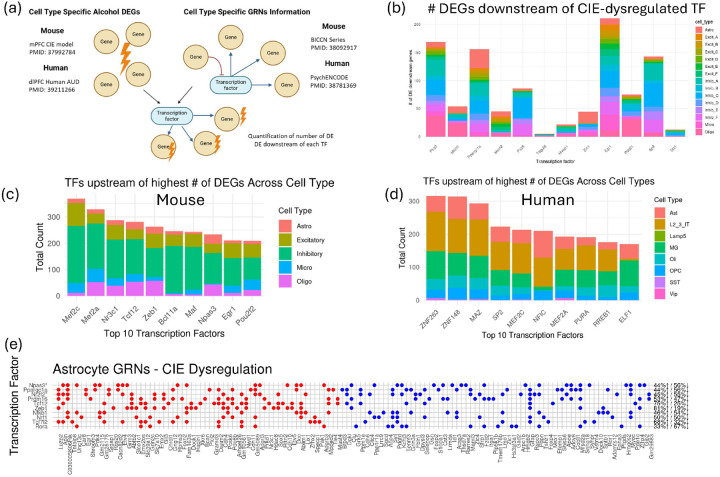
Identification of upstream regulators of CIE- and AUD-dysregulated genes. **(a)** Workflow illustrating the integration of gene regulatory network (GRN) information with CIE- and AUD-dysregulated genes, created in BioRender. Salem, N. (2026) https://BioRender.com/j581e31. **(b)** Bar chart showing the number of CIE differentially expressed genes (CIE-DEGs) downstream of each transcription factor (TF, y-axis). TFs (x-axis) are ranked in descending order based on the number of cell types in which they are dysregulated. Only TFs dysregulated in the highest number of cell types are shown. **(c–d)** Number of dysregulated genes downstream of each TF in (c) CIE and (d) AUD datasets. TFs are ordered by the total number of dysregulated downstream genes. Bars are color-coded by the cell type in which the downstream genes are dysregulated. **(e)** Dot plot showing astrocyte CIE-DEGs (x-axis) and their upstream TFs (y-axis). A dot indicates that the gene on the x-axis is a downstream target of the corresponding TF. Red dots represent upregulated genes; blue dots represent downregulated genes. The proportion of upregulated and downregulated downstream genes is indicated for each TF.

To characterize gene regulatory networks (GRNs) encompassing the DEGs in the mouse and human datasets, we leveraged cell-type-specific gene regulatory network information provided by the NIH’s Brain Research through Advancing Innovative Neurotechnologies (BRAIN) Initiative - Cell Census Network (BICCN)^17^ for mouse GRNs and the PsychENCODE consortium for human brain GRNs^6^. We mapped our cell types to the corresponding reference GRN cell types and imported lists of TFs and their downstream target genes. For each of the mouse and human datasets, we (1) identified alcohol-dysregulated TFs, (2) determined the number of alcohol-dysregulated genes downstream of each TF, and (3) identified TFs upstream of each alcohol-dysregulated gene (Figure 3a).

#### Mouse Alcohol Dysregulated Gene Regulatory Networks

We identified the TFs Pbx3, Eomes, Meis1, and Ppargc1a to be downregulated in most cell types in the alcohol-dependent group. The TFs, Hivep1, Egr1, and Celf5 were upregulated across multiple cell types in the alcohol-dependent group. We assessed whether dysregulation of these TFs corresponded with dysregulation of their downstream genes by comparing the overlap of differentially expressed TF (DE-TFs) and their DEGs. DE-TFs, Egr1, Ppargc1a, Pbx3, Sp8, and Pax6, were upstream regulators of 100–200 DEGs across cell types (Figure 3b). Downstream genes of Eomes, although a DE-TF, were not differentially expressed in alcohol-dependent samples.

We then aimed to identify TFs upstream of majority of DEGs in each cell type. In astrocytes, upregulated TF Npas3 regulated 36 dysregulated genes, while the downregulated TF Nr2f2 regulated 33 DEGs (Figure 3e). Tcf12, Zeb1, Tcf712, and Klf7 were upstream of predominantly upregulated genes, whereas Mef2a was upstream of predominantly downregulated genes (**Supplementary Table 4**). In microglia, most DEGs were upregulated in alcohol-dependent samples. The upregulated TFs Mef2a and Mef2c regulated 51 and 38 DEGs, respectively, while Egr1 and Mafb regulated 28 and 22 DEGs (**Supplementary Table 4**). In oligodendrocytes, Zeb1, Npas3, Nr3c1, Nfia, and Pbx3 were dysregulated TFs regulating 57, 43, 39, 37, and 37 DEGs, respectively. Tcf12 showed a bias toward upregulation (72% upregulated, 28% downregulated), while Nfatc1 regulated mostly downregulated genes (67% down, 33% up; **Supplementary Table 4**). In neuronal cells, Mef2a, Mef2c, Bcl11a, Maf, and Nr3c1 were upstream of most DEGs in inhibitory neurons; Mef2c, Egr1, Nr3c1, and Zeb1 were upstream of most DEGs in excitatory neurons. Collectively, across cell types Mef2c, Mef2a, Nr3c1 & Tcf12 were upstream of most DEGs (Figure 3c). Genes downstream of these TFs are involved in cell-cell adhesion, synapse and cell junction organization, synaptic transmission processes, and extracellular matrix organization.

#### Gene Regulatory Networks in Human Alcohol Dependence

In human AUD, ZNF263, ZNF148, and MAZ were upstream of most DEGs (Figure 3d), with most downstream gene dysregulation contributed by layer 2/3 intratelencephalic (IT) neurons. SPI1, BCL11A, BCL11B, and IRF1 regulated most DEGs in microglia; NFIC, ZNF148, MAZ, SP1, and SP2 regulated most astrocyte DEGs; ZNF148, CTCF, RREB1, MAZ, ZNF263, SOX9, and TCF12 regulated most oligodendrocyte DEGs (**Supplementary Table 4**).

To identify translationally relevant master regulators of dependence dysregulated gene networks, TFs were prioritized based on the number of downstream alcohol-dysregulated genes in the mouse and human datasets across all cell types. These TFs represent non-cell-type-specific regulators with translational relevance. TFs upstream of DEGs common to both mouse and human datasets included Mef2a, Mef2c, Jund, and Nr3c1 (Table 1). Fifteen DEGs overlapped between the mouse and human downstream genes of Mef2c. Nine of those genes are involved in regulation of Rho protein signal transduction, five are involved in neuron projection development. Thirteen DEGs overlapped between Mef2a mouse and human downstream genes, three of which are involved with the adaptive immune system (CD86, S100A1, BLNK). Twelve genes overlapped between DEGs downstream of Nr3c1, 3 of which are involved with regulation of vesicle mediated transport (EPHA3, RAB27B, RIT2), ten genes overlapped between downstream DEGs of each of Jund (three of which are involved with cell adhesion, CNTN6, PCDH15, CNTN4) and Zeb1 (three of which are involved with cell adhesion CDH10, PCDH15 & MMP16).

## Discussion:

AUD is a chronic, relapsing brain disease with limited therapeutic options. This study identifies conserved, cell-type-specific transcriptomic networks in the prefrontal cortex that are dysregulated across species in alcohol dependence, and translationally relevant global and cell-type-specific targets.

A cross-species transcriptomic framework was used to identify conserved dysregulated networks underlying alcohol dependence with translational relevance. Construction of gene co-expression modules and GRNs in mouse models of alcohol dependence and in humans with AUD enabled analysis of transcriptomic networks of alcohol dependence. Gene co-expression modules represent sets of genes with highly correlated expression patterns^21^, while GRNs comprise a collection of molecular regulators (TFs, miRNAs, and gene regulatory elements [promoters and enhancers]) that interact to regulate overall gene expression^22^. This network-level integration allows for addressing alcohol dependence as a multigene disorder. Examining these networks in a cell-type-specific context enabled us to differentiate between cell-type-agnostic and cell-type-specific mechanisms, with a focus on the PFC, a key region for executive function and reward circuitry^23–25^. Here, we integrated cell-type-specific transcriptomic studies of alcohol dependence^9,11^. We investigated cross-species alcohol-dependence-related changes in PFC transcriptomic networks by identifying (1) convergent, cell-type-specific gene co-expression modules dysregulated in both mouse and human alcohol dependence, and (2) upstream regulators of DEGs within each cell type that may represent targetable mechanisms underlying network dysregulation.

First, we identified convergent cell types between the mouse and human PFC through anchor-based integration of the two datasets. This integration revealed convergence in most excitatory and inhibitory cell types between mouse and human PFC, although certain neuronal subclusters were unique to the mouse. Notably, we identified a human AUD-specific microglial subpopulation that lacks a mouse homolog and shows enrichment for immune and cytokine signaling, and disease associated microglia genes. This divergence may reflect differences in model exposure, regional specificity, or species-specific microglial responses. Disease-associated microglia have been implicated in neurodegenerative disorders^26–28^, suggesting a potentially protective role; however, their role in AUD warrants further investigation.

We then identified cross-species alcohol-dysregulated gene co-expression modules in convergent mouse and human cell types. Modules were prioritized if they shared hub genes, demonstrated significant overlap in gene membership, and were significantly dysregulated in alcohol-dependent groups. Using these criteria, we identified an upregulated astrocytic module in both mouse and human datasets with the Gpc5 & Slc1a3 hub genes. In a secondary analysis of snRNA-seq of rat amygdala in alcohol withdrawal^8^, we identified an alcohol withdrawal-upregulated Slc1a3 module; this module showed a significant overlap of gene members with the mouse and human Gpc5/Slc1a3 modules. Slc1a3 encodes GLAST, a major glial glutamate transporter, with glutamate signaling known to mediate alcohol’s effects^29^. Manipulation of glutamate systems in the PFC alters addiction-related behaviors^30^, and GLAST deletion reduces alcohol consumption and reward^31^. The cross-species cross-dependence models’ conservation of this module underscores a conserved astrocytic mechanism and a translationally relevant mechanistic target.

Similarly, we identified a mouse-human conserved, dependence-upregulated module in oligodendrocytes, with PDE4B as a highly connected gene in the mouse and human modules. Overlapping genes in these modules are involved in intracellular signaling and cell junction assembly. PDE4B, among nineteen independent single-nucleotide polymorphisms that were genome-wide significant, is associated with addiction risk factors across multiple ancestries in a multivariate genome-wide association meta-analysis^32^. Various non-selective PDE4 inhibitors have been shown to decrease ethanol intake in rodents^33–36^. Selective Pde4b inhibition did not decrease ethanol intake in mice^37^. Apremilast, a nonselective PDE4 inhibitor with reduced side effects, reduced drinking in various animal models^38–42^ and in non-treatment-seeking individuals with AUD^41^. This convergence of human genetics, mouse-human transcriptomic data, and pharmacological studies highlights PDE4B and its co-expression network as a conserved molecular pathway contributing to alcohol dependence and identifies oligodendrocytes as a key cell type in these mechanisms.

We further investigated whether DEGs in each cell type are regulated by common TFs, potentially acting as master regulators of alcohol dependence-related gene expression. Identification of such regulators offers novel avenues for intervention. Previous research has shown that manipulating GRNs can modulate addiction-related gene expression and behaviors^43–46^. Alcohol dependence can disrupt GRNs by altering TF regulatory potential or network structure^47^. While cell-type-specific GRN profiling in alcohol dependence is limited, we utilized publicly available networks from control datasets to identify TFs upstream of DEGs. This approach revealed a set of TFs (Mef2a, Mef2c, Jund, Nr3c1, and Zeb1) as global regulators of dependence-associated networks. NR3C1 (glucocorticoid receptor) is a key regulator of alcohol-related behaviors, with genetic and epigenetic alterations linked to early alcohol use and AUD^48–51^. JunD modulates oxidative stress and neuroprotection^52–54^, while Mef2 factors regulate synaptic plasticity and are implicated in addiction^44,55^; MEF2C is an activity-dependent transcription factor that regulates synaptic homeostasis^56,57^, while also coordinating transcriptional programs across neuronal and immune lineages^58^. MEF2C dysregulation in both neuronal and neuroimmune populations leads to aberrant synaptic remodeling, impaired learning and memory, and behavioral phenotypes relevant to neurodevelopmental and neuropsychiatric vulnerability^57–60^. Disruption of MEF2C-regulated gene networks may therefore represent a convergent mechanism linking maladaptive activity-dependent synaptic plasticity with neuroimmune dysregulation that promotes dependence-associated circuit instability. Zeb1 has been identified as a regulator in cocaine use disorder^61^. These TFs represent promising targets for modulating dysregulated gene networks across cell types in AUD. Overall, this cross-species integration study identified conserved cell-type-specific networks and upstream regulators dysregulated in alcohol dependence. The identification of conserved dysregulated signatures across species and the complexity of dependence in individuals with AUDs underscore the generalizability of these mechanisms and highlight new opportunities for therapeutic intervention. This work provides prioritized regulatory targets and a foundation for mechanistic studies to test the roles of conserved gene networks and upstream regulators in alcohol dependence. Future studies aim at multiple parallel perturbations of genes within dysregulated modules, and targeted manipulation of key transcription factors will be essential for determining how these regulatory programs mediate alcohol-related behaviors.

## Supplementary Material

Supplementary Files

This is a list of supplementary files associated with this preprint. Click to download.

- Supplementarytable1conservedmodules.xlsx
- Supplementarytable2.xlsx
- Supplementarytable3GRNmousedotplots.xlsx
- Supplementarytable4GRNhumandotplots.xlsx

## Figures and Tables

**Table 1:** TFs upstream of the majority of DEGs in mouse and human datasets. The table shows the number of DEGs in each of the mouse and human datasets, as well as the number of overlapping downstream DEGs.

TF (human symbol)	# of downstream AUD DEGs	TF (mouse symbol)	# of downstream CIE DEGs	# of overlapping DEGs	Overlapping DEGs
MEF2C	213	Mef2c	369	15	DYNC1l1, ETV, EPHA5, MMP16, OLFM3, RIT2, ARHGAP25, CST3, TENM1, DOCK10, MPED2, S100A1, APOE, CD86, MITF
MEF2A	193	Mef2a	329	13	ATP1B2, DYNC1l1, ZFPM2, MMP16, PTCHD4, EPHA5, OLFM3, ARHGAP25, S100A1, SYN3, APOE, BLNK, CD86
NR3C1	150	Nr3c1	287	12	ZFPM2, EPHA3, GOLIM4, MMP16, OLFM3, DHRS3, RIT2, RAB27B, S100A1, SYN3, MITF, SLC9A3R1
JUND	146	Jund	200	10	CNTN4, ZFPM2, RAB27B, CNTN6, OLFM3, MARCKSL1, PCDH15, CST3, GRIK1, GRM3
ZEB1	88	Zeb1	263	10	KCND2, LUZP2, MMP16, PAM, ZFPM2, CDH10, PRKD1, FGF12, MPPED2, PCDH15

## References

[1] Mignogna K. M., Bacanu S. A., Riley B. P., Wolen A. R. & Miles M. F. Cross-species alcohol dependence-associated gene networks: Co-analysis of mouse brain gene expression and human genome-wide association data. PLOS ONE 14, e0202063 (2019). 31017905 10.1371/journal.pone.0202063 PMC6481773

[2] Huggett S. B., Bubier J. A., Chesler E. J. & Palmer R. H. C. Do Meso-Limbic Gene Expression Findings from Mouse Models of Cocaine Self-Administration Recapitulate Human Cocaine Use Disorder? Genes, brain, and behavior 20, e12689 (2020). 32720468 10.1111/gbb.12689 PMC8173322

[3] Mews P. Convergent abnormalities in striatal gene networks in human cocaine use disorder and mouse cocaine administration models. Science Advances 9, eadd8946 (2023). 36763659 10.1126/sciadv.add8946 PMC9916993

[4] Ogenpohl J. W. Cross-Species Co-analysis of Prefrontal Cortex Chronic Ethanol Transcriptome Responses in Mice and Monkeys. Frontiers in Molecular Neuroscience 12, (2019). 10.3389/fnmol.2019.00197 PMC6701453 31456662

[5] Lorrai I. Gene network inference and master regulator analysis identifies the estrogen-related receptor gamma (ERRγ) as a therapeutic target for alcohol use disorder (AUD). bioRxivorg (2025) doi:10.1101/2025.11.15.688629.

[6] Emani P. S. Single-cell genomics and regulatory networks for 388 human brains. Science 384, eadi5199 (2024). 38781369 10.1126/science.adi5199 PMC11365579

[7] Brenner E. Single cell transcriptome profiling of the human alcohol-dependent brain. Hum. Mol. Genet. 29, 1144–1153 (2020). 32142123 10.1093/hmg/ddaa038 PMC7206851

[8] Dilly G. A., Kittleman C. W., Kerr T. M., Messing R. O. & Mayfield R. D. Cell-type specific changes in PKC-delta neurons of the central amygdala during alcohol withdrawal. Transl. Psychiatry 12, 289 (2022). 35859068 10.1038/s41398-022-02063-0 PMC9300707

[9] Salem N. A. Cell-type brain-region specific changes in prefrontal cortex of a mouse model of alcohol dependence. Neurobiol. Dis. 190, 106361 (2023). 37992784 10.1016/j.nbd.2023.106361 PMC10874299

[10] van den Oord E. J. C. G., Xie L. Y., Zhao M., Aberg K. A. & Clark S. L. A single-nucleus transcriptomics study of alcohol use disorder in the nucleus accumbens. Addict. Biol. 28, e13250 (2023). 36577731 10.1111/adb.13250

[11] Warden A. S. Integrative genomics approach identifies glial transcriptomic dysregulation and risk in the cortex of individuals with Alcohol Use Disorder. Biol. Psychiatry (2025) doi:10.1016/j.biopsych.2025.02.895. PMC12667600 40024496

[12] Erickson E. K. Cortical astrocytes regulate ethanol consumption and intoxication in mice. Neuropsychopharmacology 46, 500–508 (2021). 32464636 10.1038/s41386-020-0721-0 PMC8027025

[13] Warden A. S. Microglia control escalation of drinking in alcohol-dependent mice: Genomic and synaptic drivers. Biol. Psychiatry 88, 910–921 (2020). 32680583 10.1016/j.biopsych.2020.05.011 PMC7674270

[14] Warden A. S. Microglia depletion and alcohol: Transcriptome and behavioral profiles. Addict. Biol. 26, e12889 (2021). 32176824 10.1111/adb.12889 PMC8510547

[15] Stuart T. Comprehensive Integration of Single-Cell Data. Cell 177, 1888–1902.e21 (2019). 31178118 10.1016/j.cell.2019.05.031 PMC6687398

[16] Morabito S., Reese F., Rahimzadeh N., Miyoshi E. & Swarup V. hdWGCNA identifies co-expression networks in high-dimensional transcriptomics data. Cell Rep. Methods 3, 100498 (2023). 37426759 10.1016/j.crmeth.2023.100498 PMC10326379

[17] Zu S. Single-cell analysis of chromatin accessibility in the adult mouse brain. Nature 624, 378–389 (2023). 38092917 10.1038/s41586-023-06824-9 PMC10719105

[18] Berto S. Accelerated evolution of oligodendrocytes in the human brain. Proceedings of the National Academy of Sciences of the United States of America 116, 24334–24342 (2019). 31712436 10.1073/pnas.1907982116 PMC6883816

[19] Steel G. & Lutz E. M. Characterisation of the mouse vasoactive intestinal peptide receptor type 2 gene, Vipr2, and identification of a polymorphic LINE-1-like sequence that confers altered promoter activity. J. Neuroendocrinol. 19, 14–25 (2007). 17184482 10.1111/j.1365-2826.2006.01498.x PMC1804204

[20] Feltri M. L., Suter U. & Relvas J. B. The function of RhoGTPases in axon ensheathment and myelination. Glia 56, 1508–1517 (2008). 18803320 10.1002/glia.20752 PMC2615182

[21] Stuart J. M., Segal E., Koller D. & Kim S. K. A gene-coexpression network for global discovery of conserved genetic modules. Science 302, 249–255 (2003). 12934013 10.1126/science.1087447

[22] Davidson E. & Levin M. Gene regulatory networks. Proc. Natl. Acad. Sci. U. S. A. 102, 4935 (2005). 15809445 10.1073/pnas.0502024102 PMC556010

[23] Ball G. Executive functions and prefrontal cortex: a matter of persistence? Front Syst Neurosci 5, 3 (2011). 21286223 10.3389/fnsys.2011.00003 PMC3031025

[24] Abernathy K., Chandler L. J. & Woodward J. J. Alcohol and the prefrontal cortex. Int. Rev. Neurobiol. 91, 289–320 (2010). 20813246 10.1016/S0074-7742(10)91009-X PMC3593065

[25] Heilig M. Reprogramming of mPFC transcriptome and function in alcohol dependence. Genes Brain Behav. 16, 86–100 (2017). 27657733 10.1111/gbb.12344 PMC5555395

[26] Deczkowska A. Disease-associated microglia: A universal immune sensor of neurodegeneration. Cell 173, 1073–1081 (2018). 29775591 10.1016/j.cell.2018.05.003

[27] Keren-Shaul H. A Unique Microglia Type Associated with Restricting Development of Alzheimer’s Disease. Cell 169, 1276–1290.e17 (2017). 28602351 10.1016/j.cell.2017.05.018

[28] Silvin A. Dual ontogeny of disease-associated microglia and disease inflammatory macrophages in aging and neurodegeneration. Immunity 55, 1448–1465.e6 (2022). 35931085 10.1016/j.immuni.2022.07.004

[29] Gonzales R. A. & Jaworski J. N. Alcohol and glutamate. Alcohol Health Res. World 21, 120–127 (1997). 15704347 PMC6826830

[30] Moghaddam B. Stress activation of glutamate neurotransmission in the prefrontal cortex: implications for dopamine-associated psychiatric disorders. Biol. Psychiatry 51, 775–787 (2002). 12007451 10.1016/s0006-3223(01)01362-2

[31] Karlsson R.-M. Reduced alcohol intake and reward associated with impaired endocannabinoid signaling in mice with a deletion of the glutamate transporter GLAST. Neuropharmacology 63, 181–189 (2012). 22342743 10.1016/j.neuropharm.2012.01.027 PMC3372600

[32] Hatoum A. S. Multivariate genome-wide association meta-analysis of over 1 million subjects identifies loci underlying multiple substance use disorders. Nature Mental Health 1, 210–223 (2023). 37250466 10.1038/s44220-023-00034-y PMC10217792

[33] Blednov Y. A., Benavidez J. M., Black M. & Harris R. A. Inhibition of phosphodiesterase 4 reduces ethanol intake and preference in C57BL/6J mice. Front. Neurosci. 8, 129 (2014). 24904269 10.3389/fnins.2014.00129 PMC4034339

[34] Wen R.-T. The phosphodiesterase-4 (PDE4) inhibitor rolipram decreases ethanol seeking and consumption in alcohol-preferring Fawn-Hooded rats. Alcohol. Clin. Exp. Res. 36, 2157–2167 (2012). 22671516 10.1111/j.1530-0277.2012.01845.x PMC4335658

[35] Franklin K. M. Reduction of alcohol drinking of alcohol-preferring (P) and high-alcohol drinking (HAD1) rats by targeting phosphodiesterase-4 (PDE4). Psychopharmacology 232, 2251–2262 (2015). 25585681 10.1007/s00213-014-3852-3 PMC4465875

[36] Rutten K. Enhanced long-term depression and impaired reversal learning in phosphodiesterase 4B-knockout (PDE4B−/−) mice. Neuropharmacology 61, 138–147 (2011). 21458469 10.1016/j.neuropharm.2011.03.020

[37] Blednov Y. A., Da Costa A., Mason S., Mayfield J. & Messing R. O. Selective PDE4B and PDE4D inhibitors produce distinct behavioral responses to ethanol and GABAergic drugs in mice. Neuropharmacology 231, 109508 (2023). 36935006 10.1016/j.neuropharm.2023.109508 PMC10127528

[38] Blednov Y. A., Da Costa A. J., Harris R. A. & Messing R. O. Apremilast alters behavioral responses to ethanol in mice: II. Increased sedation, intoxication, and reduced acute functional tolerance. Alcohol. Clin. Exp. Res. 42, 939–951 (2018). 29469954 10.1111/acer.13615 PMC5916327

[39] Blednov Y. A. Apremilast regulates acute effects of ethanol and other GABAergic drugs via protein kinase A-dependent signaling. Neuropharmacology 178, 108220 (2020). 32736086 10.1016/j.neuropharm.2020.108220 PMC7544627

[40] Blednov Y. A. Apremilast-induced increases in acute ethanol intoxication and decreases in ethanol drinking in mice involve PKA phosphorylation of GABAA β3 subunits. Neuropharmacology 220, 109255 (2022). 36152689 10.1016/j.neuropharm.2022.109255 PMC9810330

[41] Grigsby K. B. Preclinical and clinical evidence for suppression of alcohol intake by apremilast. J. Clin. Invest. 133, (2023). 10.1172/JCI159103 PMC10014105 36656645

[42] Vozella V. Apremilast reduces co-occurring alcohol drinking and mechanical allodynia and regulates central amygdala GABAergic transmission. JCI Insight 10, (2025). 10.1172/jci.insight.189732 PMC12016922 40260918

[43] McClung C. A. & Nestler E. J. Regulation of gene expression and cocaine reward by CREB and DeltaFosB. Nat. Neurosci. 6, 1208–1215 (2003). 14566342 10.1038/nn1143

[44] Pulipparacharuvil S. Cocaine regulates MEF2 to control synaptic and behavioral plasticity. Neuron 59, 621–633 (2008). 18760698 10.1016/j.neuron.2008.06.020 PMC2626175

[45] Renthal W. Genome-wide analysis of chromatin regulation by cocaine reveals a role for sirtuins. Neuron 62, 335–348 (2009). 19447090 10.1016/j.neuron.2009.03.026 PMC2779727

[46] Savell K. E. A dopamine-induced gene expression signature regulates neuronal function and cocaine response. Sci. Adv. 6, eaba4221 (2020). 32637607 10.1126/sciadv.aba4221 PMC7314536

[47] Duren Z. Sc-compReg enables the comparison of gene regulatory networks between conditions using single-cell data. Nat. Commun. 12, 4763 (2021). 34362918 10.1038/s41467-021-25089-2 PMC8346476

[48] Desrivières S. Glucocorticoid receptor (NR3C1) gene polymorphisms and onset of alcohol abuse in adolescents. Addict. Biol. 16, 510–513 (2011). 20731635 10.1111/j.1369-1600.2010.00239.x PMC3428936

[49] Gatta E. Genome-wide methylation in alcohol use disorder subjects: implications for an epigenetic regulation of the cortico-limbic glucocorticoid receptors (NR3C1). Mol. Psychiatry 26, 1029–1041 (2021). 31239533 10.1038/s41380-019-0449-6 PMC6930366

[50] Repunte-Canonigo V. Identifying candidate drivers of alcohol dependence-induced excessive drinking by assembly and interrogation of brain-specific regulatory networks. Genome Biol. 16, (2015). 10.1186/s13059-015-0593-5 PMC4410476 25886852

[51] Park S. NR3C1-mediated epigenetic regulation suppresses astrocytic immune responses in mice. Nat. Commun. 16, 8330 (2025). 40983615 10.1038/s41467-025-64088-5 PMC12454645

[52] Hull R. P. Combined ChIP-Seq and transcriptome analysis identifies AP-1/JunD as a primary regulator of oxidative stress and IL-1β synthesis in macrophages. BMC Genomics 14, 92 (2013). 23398888 10.1186/1471-2164-14-92 PMC3608227

[53] Paneni F. Deletion of the activated protein-1 transcription factor JunD induces oxidative stress and accelerates age-related endothelial dysfunction. Circulation 127, 1229–40, e1–21 (2013). 23410942 10.1161/CIRCULATIONAHA.112.000826

[54] Diaz-Cañestro C. AP-1 (activated protein-1) transcription factor JunD regulates ischemia/reperfusion brain damage via IL-1β (interleukin-1β). Stroke 50, 469–477 (2019). 30626291 10.1161/STROKEAHA.118.023739

[55] Dietrich J.-B., Takemori H., Grosch-Dirrig S., Bertorello A. & Zwiller J. Cocaine induces the expression of MEF2C transcription factor in rat striatum through activation of SIK1 and phosphorylation of the histone deacetylase HDAC5. Synapse 66, 61–70 (2012). 21954104 10.1002/syn.20988

[56] Flavell S. W. Genome-wide analysis of MEF2 transcriptional program reveals synaptic target genes and neuronal activity-dependent polyadenylation site selection. Neuron 60, 1022–1038 (2008). 19109909 10.1016/j.neuron.2008.11.029 PMC2630178

[57] Barbosa A. C. MEF2C, a transcription factor that facilitates learning and memory by negative regulation of synapse numbers and function. Proc. Natl. Acad. Sci. U. S. A. 105, 9391–9396 (2008). 18599438 10.1073/pnas.0802679105 PMC2453723

[58] Harrington A. J. MEF2C hypofunction in neuronal and neuroimmune populations produces MEF2C haploinsufficiency syndrome-like behaviors in mice. Biol. Psychiatry 88, 488–499 (2020). 32418612 10.1016/j.biopsych.2020.03.011 PMC7483399

[59] Harrington A. J. MEF2C regulates cortical inhibitory and excitatory synapses and behaviors relevant to neurodevelopmental disorders. Elife 5, (2016). 10.7554/eLife.20059 PMC5094851 27779093

[60] Tu S. NitroSynapsin therapy for a mouse MEF2C haploinsufficiency model of human autism. Nat. Commun. 8, 1488 (2017). 29133852 10.1038/s41467-017-01563-8 PMC5684358

[61] Zillich L. Cell type-specific multi-omics analysis of cocaine use disorder in the human caudate nucleus. Nat. Commun. 16, 3381 (2025). 40204703 10.1038/s41467-025-57339-y PMC11982542

